# Development of a Multiplex TaqMan Real-Time PCR Assay for the Simultaneous Detection and Differentiation of Three Pathogenic *Yersinia* Species

**DOI:** 10.3390/vetsci13060520

**Published:** 2026-05-27

**Authors:** Xue Yang, Xin Lei, Yongjun Luo, Jiali Wang, Qiuyu Fan, Xiaofang Yan, Haohong Zheng, Ciren Zhuoma, Yunhan Zhou, Haifeng Liu, Ziyao Zhou, Zhijun Zhong, Jialiang Xin, Zhengli Chen, Guangneng Peng

**Affiliations:** 1 Key Laboratory of Animal Disease and Human Health of Sichuan, College of Veterinary Medicine, Sichuan Agricultural University, Chengdu 611130, China; yangx00112@163.com (X.Y.); 15680780217@163.com (X.L.); 410140017@163.com (H.L.); zzhou@sicau.edu.cn (Z.Z.); zhongzhijun488@126.com (Z.Z.); 2 Xinjiang Uygur Autonomous Region Center for Disease Control and Prevention, Urumqi 830002, China; luoyj8686@163.com; 3 Shuanghu County Agricultural and Animal Husbandry Science and Technology Service Station, Nagqu 853300, China

**Keywords:** *Yersinia* spp., multiplex real-time PCR, TaqMan assay, pathogen detection, wildlife surveillance

## Abstract

Three pathogenic species of the genus *Yersinia*, including Plague-associated *Yersinia pestis*, *Yersinia pseudotuberculosis*, and *Yersinia enterocolitica*, are commonly associated with human infection. Current qPCR detection methods are mainly limited to the identification of one or two *Yersinia* species in a single reaction tube, while multiplex assays for multiple genera have been more commonly reported. Therefore, the present study aimed to establish a multiplex TaqMan qPCR assay for the simultaneous detection of these three pathogenic *Yersinia* species. Primer and probe sets were designed based on the *inv* gene for *Y. pseudotuberculosis*, the *caf1* gene for *Y. pestis*, and the *foxA* gene for *Y. enterocolitica*. Under the optimized reaction conditions, the standard curve slopes for the *caf1*, *inv*, and *foxA* genes were −3.046, −2.968, and −2.948, respectively. The correlation coefficients (R^2^) ranged from 0.993 to 0.996, while the amplification efficiencies ranged from 109% to 115%. The limits of detection (LOD) were determined to be 5 × 10^2^ copies/μL for *inv* (FAM), 1 × 10^1^ copies/μL for *caf1* (ROX), and 1 × 10^1^ copies/μL for *foxA* (CY5). The sensitivity of the multiplex qPCR assay was 10- to 100-fold higher than that of conventional PCR, depending on the target. Specificity experiments demonstrated that no cross-reactivity was observed with non-target bacteria, including *Francisella tularensis*, *Brucella* spp., *Vibrio cholerae*, *Salmonella* Typhi, and *Shigella* spp. The intra-assay coefficients of variation (CVs) ranged from 0.13% to 0.79%, whereas the inter-assay CVs ranged from 0.62% to 2.61%. Among 173 spleen samples collected from wild rodents, no positive signal for *Y. pestis* or *Y. pseudotuberculosis* was detected. In contrast, *Y. enterocolitica* was detected in three samples (1.73%, 3/173). In conclusion, the multiplex qPCR assay developed in this study provides a sensitive and specific tool for the simultaneous detection of three pathogenic *Yersinia* species and has the potential to improve detection efficiency in clinical and epidemiological investigations.

## Simple Summary

*Yersinia pestis*, *Yersinia enterocolitica*, and *Yersinia pseudotuberculosis* are important pathogenic species within the genus *Yersinia*. Currently available detection methods are generally limited to identifying only one or two of these pathogens in a single reaction. In this study, we developed a multiplex TaqMan assay capable of simultaneously detecting all three pathogens in a single tube. The assay demonstrated high sensitivity, specificity, reproducibility, and good clinical applicability. This method improves the efficiency of *Yersinia* detection and provides a practical tool for clinical diagnosis and surveillance.

## 1. Introduction

The genus *Yersinia* belongs to the family Enterobacteriaceae and consists of Gram-negative bacteria. Among this genus, *Yersinia pestis*, *Yersinia pseudotuberculosis*, and *Yersinia enterocolitica* are recognized as major human pathogens. These species are considered typical zoonotic pathogens and are associated with risks to public health, food safety, and animal health [1].

*Yersinia pestis* is the causative agent of plague and is mainly transmitted among rodents and their fleas. Historically, plague was responsible for three major pandemics and caused millions of deaths worldwide [2,3]. Although large outbreaks have become uncommon in recent decades, the pathogen is still maintained in wildlife reservoirs, especially rodent populations, where continuous circulation has been reported [4]. In contrast, *Y. enterocolitica* is commonly detected in the intestines of pigs [5]. The bacterium has also been identified in rodents, which may contribute to its maintenance in natural environments [6]. Human infection is mainly associated with contaminated food [7,8]. In addition, the organism can grow at low temperatures, which may facilitate its environmental persistence [9]. *Y. pseudotuberculosis* has a broad host range that includes rodents, birds, primates, and ruminants. Outbreaks with high mortality have been reported in some animal populations [10]. Infected hosts often develop acute septicemia or chronic wasting disease, which may affect animal production and wildlife populations [11]. Epidemiological studies have shown that yersiniosis is the fourth most frequently reported zoonotic disease worldwide [12].

Notably, these three pathogenic *Yersinia* species can all be carried by rodents in natural environments. Their transmission is maintained through complex ecological cycles involving multiple hosts, vectors, and transmission pathways, resulting in stable natural foci [13,14]. The existence of shared reservoirs may increase the possibility of co-circulation and co-infection [15]. Consequently, accurate differential detection is essential for effective surveillance. In addition, *Y. pestis* is classified as a Class A notifiable infectious agent [16]. Because of the strict biosafety requirements and the risks associated with bacterial isolation and culture [17], molecular detection methods are more commonly applied in routine practice [18].

Among currently available molecular methods, real-time quantitative PCR (qPCR) is widely used because of its high sensitivity and specificity [19]. However, a limitation still exists in the detection of pathogenic *Yersinia* species. To date, only limited methods have been reported that allow the simultaneous detection and differentiation of *Y. pestis*, *Y. pseudotuberculosis*, and *Y. enterocolitica* within a single reaction. Most currently available assays are limited to single-target or dual-target detection formats [20,21,22,23,24]. From the perspective of public health surveillance, the development of a unified assay for simultaneous detection of these three pathogens is of practical importance. Laboratories involved in clinical diagnosis, food safety monitoring, and environmental surveillance often need to screen different types of samples for multiple *Yersinia* species. Compared with single-target detection strategies, multiplex assays reduce reagent consumption and labor, shorten operational time, and increase laboratory throughput during routine surveillance and outbreak investigations.

In this study, we developed a single-tube multiplex TaqMan real-time PCR assay using species-specific genes from three pathogenic *Yersinia* species. Reaction conditions were systematically optimized for the simultaneous detection and differentiation of these pathogens. We evaluated the performance of the assay in terms of sensitivity, specificity, and reproducibility. Moreover, the assay was also applied to field-collected rodent samples to assess clinical applicability.

## 2. Materials and Methods

### 2.1. Bacterial Strains

Reference strains of *Y. pestis* (EV strain, 0614F strain, Otten strain, Tjiusidej (R) strain, and Microtus strain 201), *Y. pseudotuberculosis* (CMCC 53521), and *Y. enterocolitica*. O:3 (CNCC 52203), O:8 (CNCC 52211), and O:9 (CNCC 52212) were included in this study. Non-target bacterial species used for specificity evaluation included *Francisella tularensis* (LVS strain), *Brucella* spp. (CMCC 55010), *Bacillus anthracis* (CMCC 18001), *Vibrio cholerae*, *Salmonella Typhi* (CMCC 50071), and *Shigella* spp. (CMCC 51105). All strains were obtained from the National Institute for Food and Drug Control [25].

### 2.2. Primer and Probe Design

**Figure 1:**
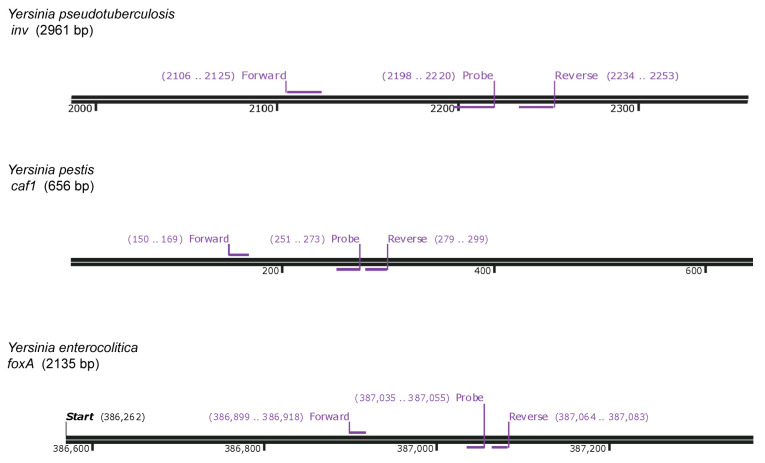
Schematic diagram of primer and probe locations within target genes. The positions of forward primers, reverse primers, and TaqMan probes are shown for the *inv* (*Yersinia pseudotuberculosis*), *caf1* (*Yersinia pestis*), and *foxA* (*Yersinia enterocolitica*) genes. The relative locations of each primer-probe set are indicated based on the gene coordinates. Numbers represent nucleotide positions within the corresponding gene sequences.

Species-specific target genes were selected for the three pathogenic *Yersinia* species, including the *caf1* gene [26] for *Y. pestis*, the *inv* gene [27] for *Y. pseudotuberculosis*, and the *foxA* gene [20] for *Y. enterocolitica*. Gene sequences were retrieved from the National Center for Biotechnology Information (NCBI) database. Sequences from different strains and geographic origins were included for comparative analysis. Multiple sequence alignment and comparative analyses were performed using SnapGene to identify conserved regions suitable for primer and probe design. Candidate regions were further analyzed using the BLAST tool in NCBI to evaluate specificity against non-target organisms (https://blast.ncbi.nlm.nih.gov/Blast.cgi (accessed on 24 May 2026)). Regions with low homology to non-target species were preferentially selected. Primers and TaqMan probes were designed using Primer Express 3.0 (Waltham, MA, USA) and synthesized by Sangon Biotech Co., Ltd. (Shanghai, China). Primer and probe sequences are listed in Table 1, and their locations within the target genes are shown in Figure 1.

### 2.3. Nucleic Acid Extraction and Preparation of Recombinant Plasmids

Genomic DNA was extracted using a commercial bacterial DNA extraction kit (Tiangen Biotech Co., Ltd., Beijing, China) according to the manufacturer’s instructions. Extracted DNA was stored at −20 °C until use. Target gene fragments (*inv*, *caf1*, and *foxA*) were amplified and cloned into the pESY-T vector to generate recombinant plasmids pESY-T-I9, pESY-T-C4, and pESY-T-F4. The recombinant constructs were verified by Sanger sequencing and subsequently used as quantitative standards.

### 2.4. Optimization of the Multiplex Reaction System

Real-time PCR was performed in a total reaction volume of 20 μL, containing 10 μL of 2× Premix Ex Taq™ (Probe qPCR). Primers (10 μM) were added at 0.4 μL each for the forward and reverse reactions, and probes (10 μM) were added at volumes ranging from 0.2 to 1.0 μL per reaction. Nuclease-free water was added to adjust the final reaction volume.

The annealing temperature was optimized using a gradient ranging from 52 °C to 58 °C (52 °C, 54 °C, 55 °C, 56 °C, and 58 °C). Amplification was performed for 45 cycles, and fluorescence signals were collected during the extension phase of each cycle. All reactions were conducted in triplicate.

### 2.5. Establishment of the Standard Curve

Recombinant plasmid concentrations were measured using a NanoDrop One spectrophotometer, and copy numbers were calculated based on plasmid concentration and molecular weight. Plasmids were diluted to 1 × 10^10^ copies/μL and subsequently subjected to ten-fold serial dilutions down to 1 × 10^1^ copies/μL to generate standard templates. Each dilution was used as a template for qPCR amplification, and all reactions were performed in triplicate. Standard curves were constructed according to Ct values, and amplification efficiency, slope, and correlation coefficient (R^2^) were calculated using the instrument software (GraphPad Prism version 10.1.2 San Diego, CA, USA).

### 2.6. Specificity Evaluation

The specificity of the multiplex TaqMan real-time PCR assay was evaluated using nucleic acids extracted from recombinant plasmids and non-target bacterial species, including *Francisella tularensis*, *Brucella* spp., *Bacillus anthracis*, *Vibrio cholerae*, *Salmonella Typhi*, and *Shigella* spp. All templates were detected under optimized reaction conditions. Specificity was assessed based on the presence or absence of amplification signals.

### 2.7. Sensitivity Analysis

Serial ten-fold dilutions of recombinant plasmids ranging from 1 × 10^8^ to 1 × 10^0^ copies/μL were prepared and used as templates (1.5 μL per reaction). Nuclease-free water (ddH_2_O) was included as a negative control. Multiplex TaqMan real-time PCR and conventional PCR assays were performed under their respective optimized conditions to compare analytical sensitivity. For conventional PCR, the same primer sets (without probes) were used to ensure consistency in target regions and to allow a direct comparison between methods.

The limit of detection (LOD) was defined as the lowest concentration at which a positive amplification signal could be consistently detected. To assess potential interference among targets, the multiplex assay was performed using both individual and mixed plasmid templates. All reactions were conducted in triplicate, and amplification performance was compared.

### 2.8. Repeatability and Reproducibility Assessment

Recombinant plasmids at concentrations of 1 × 10^4^, 1 × 10^5^, and 1 × 10^6^ copies/μL were used to evaluate assay precision. For intra-assay variability, each concentration was tested in triplicate within a single run under identical conditions. For inter-assay variability, the same concentrations were tested in triplicate at one-week intervals. Nuclease-free water was included as a negative control in each experiment.

The coefficient of variation (CV) of Ct values was calculated using the following formula: CV (%) = (standard deviation of Ct values/mean Ct value) × 100%.

### 2.9. Clinical Sample Collection and Detection

To evaluate the performance of the developed assay, spleen samples [18] from wild rodents collected in different regions of China were analyzed using both conventional PCR and the established multiplex qPCR method (Table 2). A total of 173 spleen samples were collected in July 2025 from multiple provinces, including Inner Mongolia n=24, Liaoning Province n=15, Jilin Province n=19, Heilongjiang Province n=39, and Sichuan Province (Ya’an region, *n* = 76).

DNA was extracted from all samples prior to detection under identical experimental conditions. Detection results obtained using the multiplex qPCR assay were compared with those of conventional PCR to evaluate assay performance and practical applicability.

In this study, artificial intelligence (AI) tools were used to assist with English language polishing and manuscript formatting optimization during the research and paper writing process. The AI tools were only used as an aid to improve work efficiency and accuracy of expression, and all experimental design, data generation, result interpretation, key conclusion extraction, and final manuscript modification were independently completed by the authors.

## 3. Results

### 3.1. Optimization Results of Reaction Conditions

**Figure 2:**
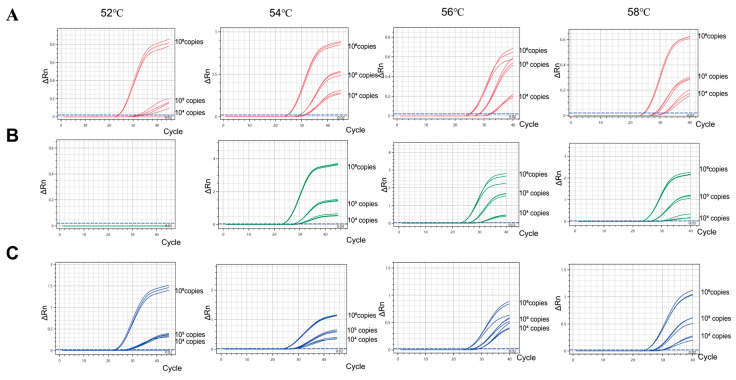
Optimization of annealing temperature for multiplex TaqMan real-time PCR assay. Amplification curves of I9, C4 and F4 targets at different annealing temperatures (52–58 °C) using serially diluted recombinant plasmid templates. (**A**) I9 target (*Yersinia pseudotuberculosis*); (**B**) C4 target (*Yersinia pestis*); (**C**) F4 target (*Yersinia enterocolitica*). Stable and consistent amplification performance of all three targets was observed at 54 °C.

The reaction conditions of the multiplex TaqMan real-time PCR assay were optimized using an orthogonal experimental design. An annealing temperature of 54 °C was identified as optimal (Figure 2). The final primer and probe concentrations were set at 0.2 μM and 0.3 μM, respectively (Table 3).

Under optimized conditions, all targets exhibited stable amplification curves with consistent fluorescence signals, confirming efficient multiplex amplification.

### 3.2. Establishment of the Standard Curve

**Figure 3:**
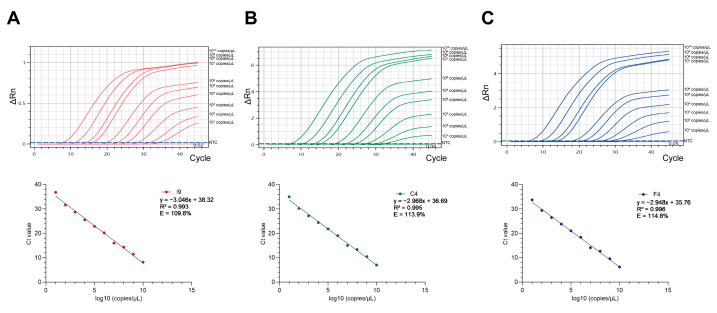
Amplification curves and standard curves of three target genes in the established multiplex TaqMan real-time PCR assay. (**A**) Amplification curve and standard curve of I9 target (*Yersinia pseudotuberculosis*); (**B**) Amplification curve and standard curve of C4 target (*Yersinia pestis*); (**C**) Amplification curve and standard curve of F4 target (*Yersinia enterocolitica*). Serial 10-fold dilutions of recombinant plasmids were used. Linear regression equation, correlation coefficient (R2) and amplification efficiency (E) are displayed for each standard curve. NTC: no template control.

Amplification curves of the three target genes (*caf1*, *inv*, and *foxA*) were generated using ten-fold serial dilutions of recombinant plasmids (10^6^–10^1^ copies/μL). As shown in Figure 3, all targets exhibited typical sigmoidal amplification curves with clear separation among different template concentrations. Based on these results, standard curves were constructed, yielding slopes of −3.046, −2.968, and −2.948 for I9, C4, and F4, respectively. The correlation coefficients (R^2^) ranged from 0.993 to 0.996, demonstrating good linearity across the tested range (Figure 3). The amplification efficiencies were calculated to be between 109% and 115%, indicating efficient amplification performance of the multiplex TaqMan real-time PCR assay.

### 3.3. Specificity of the Multiplex qPCR Assay

**Figure 4:**
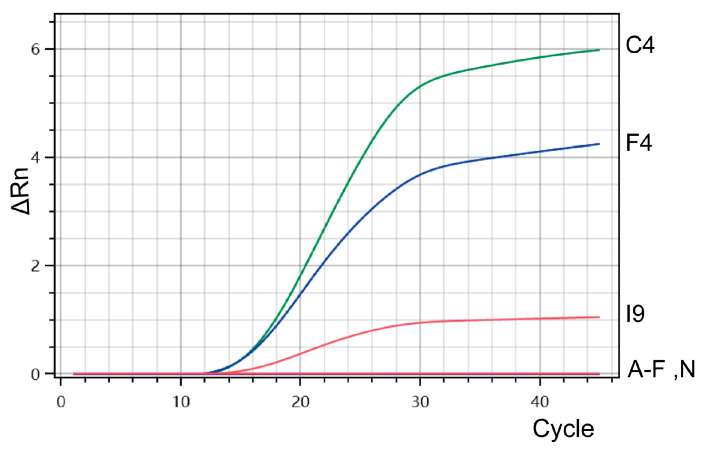
Specificity analysis of the multiplex TaqMan real-time PCR assay. Lanes A: *Francisella tularensis*; Lanes B: *Brucella* spp.; Lanes C: *Bacillus anthracis*; Lanes D: *Vibrio cholerae*; Lanes E: *Salmonella Typhi*; Lanes F: *Shigella* spp.; N: ddH_2_O (negative control); C4: *Yersinia pestis*; F4: *Yersinia enterocolitica*, I9: *Yersinia pseudotuberculosis*.

The specificity of the multiplex TaqMan real-time PCR assay was evaluated using nucleic acids from both target and non-target organisms. The results showed that specific amplification curves were observed only for the target *Yersinia* species, whereas no amplification signals were detected for non-target bacterial species or the negative control (Figure 4).

### 3.4. Sensitivity Determination of qPCR

**Figure 5:**
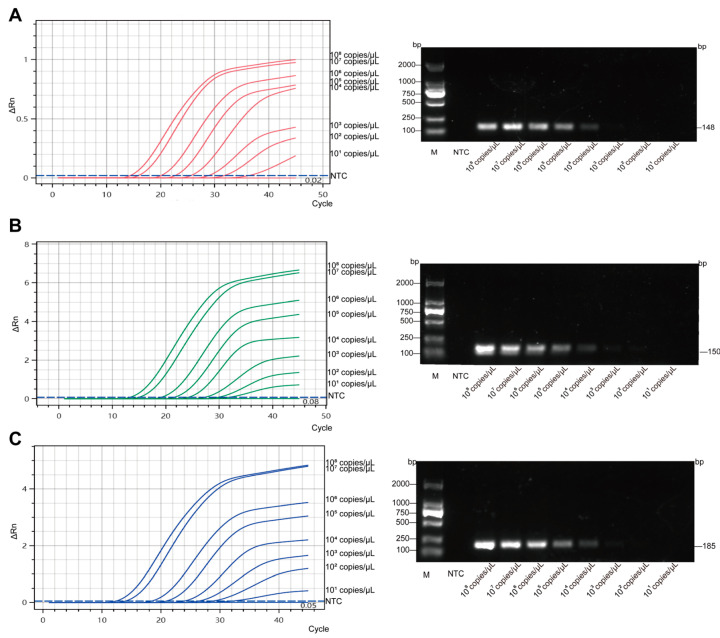
Analytical sensitivity of the multiplex TaqMan real-time PCR assay for three pathogenic *Yersinia* species. (**A**) Sensitivity detection of I9 target (*Yersinia pseudotuberculosis*, 148 bp); (**B**) Sensitivity detection of C4 target (*Yersinia pestis*, 150 bp); (**C**) Sensitivity detection of F4 target (*Yersinia enterocolitica*, 185 bp). (**Left panels**): Amplification curves of 10-fold serial diluted recombinant plasmids (10^1^–10^8^ copies/μL). (**Right panels**): Agarose gel electrophoresis verification of corresponding amplification products. M: DNA molecular weight marker; NTC: no template control.

Analytical sensitivity was evaluated using ten-fold serial dilutions of recombinant plasmids. A Ct threshold of ≤35 was applied for positive detection. The LODs were determined to be 5 × 10^2^ copies/μL for *inv* (I9) and 1 × 10^1^ copies/μL for both *caf1* (C4) and *foxA* (F4) (Figure 5). For conventional PCR, the detection limit was 1 × 10^3^ copies/μL for all targets.

### 3.5. Repeatability and Reproducibility

Repeatability and reproducibility were evaluated using plasmid concentrations of 1 × 10^4^, 1 × 10^5^, and 1 × 10^6^ copies/μL. For intra-assay variation, CV values ranged from 0.13% to 0.79% across all targets and concentrations. For inter-assay variation, CV values ranged from 0.62% to 2.61% (Table 4).

### 3.6. Evaluation Using Field-Collected Rodent Samples

**Figure 6:**
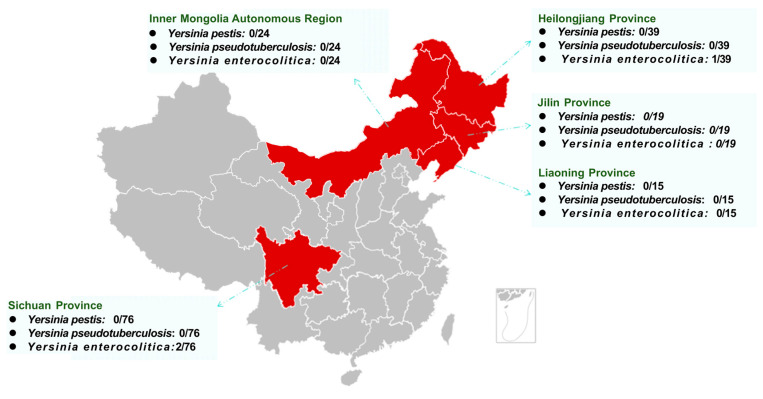
Geographic distribution and detection results of three pathogenic *Yersinia* species in wild rodent samples from five study regions in China. Red areas indicate field sampling regions; grey areas indicate non-sampling regions. Values in each region are presented as positive cases/total tested samples for *Yersinia pestis*, *Yersinia pseudotuberculosis* and *Yersinia enterocolitica*, respectively.

A total of 173 spleen samples were analyzed. No positive signals were detected for *Y. pestis* or *Y. pseudotuberculosis*. In contrast, *Y. enterocolitica* was detected in 3 samples (1.73%, 3/173), including one from Heilongjiang Province and two from Ya’an, Sichuan Province (Figure 6).

## 4. Discussion

Rapid and accurate detection of pathogenic *Yersinia* species is important for public health surveillance and outbreak control, particularly in wildlife-associated ecological systems where these pathogens show clear zoonotic characteristics and circulate among multiple host species [18,26]. Rodents are recognized as major natural reservoirs and contribute to the maintenance and transmission of pathogenic *Yersinia*. Their ecological overlap with livestock production areas and human activities may further support cross-species transmission [18]. Such ecological interactions facilitate the long-term persistence of these pathogens in nature and increase the possibility of co-circulation or mixed infection among different pathogenic *Yersinia* species, complicating wildlife surveillance and disease management.

In clinical practice, large-scale sample screening necessitates efficient detection workflows. A single-tube multiplex reaction addresses this need by simultaneously identifying multiple pathogens while enabling real-time monitoring, thereby substantially enhancing laboratory throughput. However, only one multiplex PCR method has been previously reported for the simultaneous detection of three pathogenic *Yersinia* species [27]. Conventional multiplex PCR assays are generally used. These assays depend on post-amplification gel electrophoresis for result interpretation. Handling amplified products increases contamination risk, limits quantitative analysis, and prolongs total detection time.

In the present study, we established a multiplex TaqMan real-time PCR assay for the rapid detection and differentiation of these three pathogenic *Yersinia* species in a single reaction. The integration of multiple specific targets into one reaction system reduced operational steps and improved detection efficiency. Our multiplex TaqMan real-time qPCR assay demonstrated stable and reliable analytical performance despite the integration of three targets. Standard curve analysis demonstrated excellent linearity over a wide dynamic range (R^2^ = 0.993–0.996). Compared with earlier singleplex TaqMan assays targeting *Y. enterocolitica* [19,28], our multiplex system maintained high amplification efficiency and excellent linearity despite the incorporation of three primer–probe sets into a single reaction. Although the amplification efficiencies obtained in this study (109–115%) were slightly above the theoretical optimal range [29], similar phenomena have frequently been reported in multiplex qPCR systems and are generally associated with interactions among primers and probes or differences in fluorescence calibration rather than impaired assay performance [19,30]. Importantly, we observed no obvious competitive inhibition among targets, suggesting that the primer–probe concentrations and reaction conditions were well optimized for balanced amplification.

Regarding analytical sensitivity, our multiplex TaqMan real-time qPCR assay achieved limits of detection of 5 × 10^2^ copies/μL for *inv* (I9) and *caf1* (C4), and 1 × 10^1^ copies/μL for *foxA* (F4). Previous multiplex PCR and qPCR assays for pathogenic *Yersinia* generally reported detection limits ranging from 10^1^ to 10^3^ CFU per reaction [31]. Under ideal conditions, 1 CFU corresponds approximately to 1 genome copy. Therefore, the sensitivity achieved for the *foxA* (F4) target was comparable to the higher-performing assays reported previously, while the detection limits for *inv* (I9) and *caf1* (C4) remained within the commonly accepted range for multiplex diagnostic assays.

For precision assessment, intra-assay CVs of 0.13–0.79% and inter-assay CVs of 0.62–2.61% were observed. These values met the recommended qPCR performance criteria [29,32]. When compared with previously reported multiplex qPCR systems, our inter-assay CVs were lower than those reported by Hodžić et al. (intra-assay: 1.2–4.5%; inter-assay: 3.7–7.2%) [33] and comparable to those described for penta-plex TaqMan qPCR assays targeting protozoan pathogens (0.22–2.13%). The intra-assay variation was similarly within the range reported for multiplex TaqMan assays used in veterinary diagnostics (1.06–4.93%) [34,35]. Overall, the precision of this method was comparable to or exceeded that of existing multiplex qPCR approaches.

We applied this method to spleen samples from wild rodents in northeastern China and Sichuan Province. Field applicability assessments remain limited in published studies on pathogenic *Yersinia* detection, which have mostly focused on single regions or domestic animals [36,37,38]. *Y. pestis* and *Y. pseudotuberculosis* were not detected in this survey. One positive sample of *Y. enterocolitica* was identified in wild rodents from Northeast China, and two positive samples were detected in wild rodents from Sichuan Province. The reliability of these findings is supported by the high assay specificity and adequate analytical sensitivity for field sample analysis. The high specificity of this method minimizes the risk of false-positive results. The detection limit was also found to be sufficient for the analysis of field samples. The absence of *Y. pseudotuberculosis* may be attributed to its relatively low prevalence in natural environments [39]. Similarly, the failure to detect *Y. pestis* is likely related to the fact that the sampling sites were not located within recognized natural plague foci in China. Previous studies have shown that *Y. pestis* is primarily distributed in specific ecological systems and exhibits pronounced geographic restriction [40]. Notably, *Y. enterocolitica* exhibits pronounced regional variation in China, with higher prevalence reported in southern and certain other regions [41]. However, given the limited sample size in this study, these findings do not support the conclusion that pathogenic *Yersinia* prevalence is low in wild rodent populations across these regions. Further surveillance with larger sample sizes is warranted to confirm this observation.

Caution should be exercised when interpreting the positive results in this study. The relatively small sample size precludes definitive conclusions about the overall prevalence of pathogenic Yersinia in the monitored areas. Previous studies have shown that *Y. enterocolitica* is present in humans, animals, and food in China, and the hosts include pigs, poultry, and rodents, with typical characteristics of multi-host transmission [37,42]. Wild rodents may act as latent reservoir hosts, maintaining the cyclical transmission of pathogenic *Yersinia* in the natural environment for an extended period. This role may increase the complexity and uncertainty of long-term prevention and control in epidemic areas.

## 5. Conclusions

The multiplex TaqMan real-time PCR assay developed in this study provides a rapid, sensitive, and specific method for the simultaneous detection of three pathogenic *Yersinia* species. This method enhances detection efficiency and provides a practical tool for clinical and epidemiological applications.

## Figures and Tables

**Table 1:** Primers and probes designed.

Target Gene	Primer ID	Direction	Sequence (5′–3′)	Amplicon Region (bp)
*caf1*	C4	Forward	ACGGCAACTCTTGTTGAACC	150
		Reverse	AGATGTGCTAGTGGTTCCTGT	
		Probe	ROX-AGCCGCCAAGAGTAAGCGTACCA-BHQ-2	
*foxA*	F4	Forward	ACCGGGATAACCCGTAACAG	185
		Reverse	TGCCCATAAATGCTGCCATC	
		Probe	CY5-CCGCACTGTGGTAACCGCCGG-BHQ-3	
*inv*	I9	Forward	CAGTTTCACCGTCTCCACAC	148
		Reverse	GCTAATACTCACCGGCACAC	
		Probe	FAM-ACTCAAGCCCTGCATCCCACTGA-BHQ-1	

**Table 2:** Geographic distribution of wild rodent spleen samples collected in this study.

City	Species						
	*Microtus gregalis*	*Apodemus agrarius*	*Rattus norvegicus*	*Myopus schisticolor*	*Tamias sibiricus*	*Microtus maximowiczii*	*Cricetulus barabensis*
Aershan	4	1					
Genhe				5			
Mohe	6	6			2		
Dandong		5					
Shenyang		10					
Hunchun		8	1			3	
Ji’an		4					
Yanji	1	2					
Fuyuan		19					
Hegang		7					
Heihe		8			2		2
Jixi		1					
Ya’an			36				
Chengdu			40				
Total	11	71	77	5	4	3	2

**Table 3:** Annealing temperature and probe concentration optimization.

Type	Probe	52 °C			54 °C			56 °C			58 °C		
		1 × 10^6^	1 × 10^5^	1 × 10^4^	1 × 10^6^	1 × 10^5^	1 × 10^4^	1 × 10^6^	1 × 10^5^	1 × 10^4^	1 × 10^6^	1 × 10^5^	1 × 10^4^
I9	0.2	23.720	35.159	31.311	24.396	27.783	30.696	24.634	27.710	31.034	24.060	27.288	31.241
F4		23.154	29.212	28.948	24.770	27.902	29.762	24.853	27.425	28.852	24.129	25.435	29.808
C4		NoCq	NoCq	NoCq	23.626	26.787	30.153	23.343	26.019	29.135	23.354	26.088	31.487
I9	0.4	23.883	34.925	32.314	24.497	29.132	31.196	23.278	28.257	32.150	24.503	28.514	31.386
F4		23.056	28.364	28.001	24.309	27.970	28.373	24.641	27.186	29.028	24.200	26.998	29.857
C4		NoCq	NoCq	NoCq	23.317	27.525	29.037	25.086	26.156	29.348	23.410	26.545	30.654
I9	0.6	24.163	35.098	32.816	24.758	29.401	30.405	24.519	28.277	31.270	24.543	28.831	31.200
F4		23.106	28.118	27.612	24.555	27.916	28.400	24.282	27.304	29.419	24.127	27.160	29.764
C4		NoCq	NoCq	NoCq	23.638	28.073	28.917	22.991	26.268	29.146	23.370	26.713	30.772
I9	0.8	24.207	35.253	36.018	24.858	27.889	30.642	25.114	28.172	28.163	24.982	28.738	32.806
F4		22.828	27.303	30.680	24.450	26.587	27.504	24.265	27.057	27.662	24.218	26.958	28.992
C4		NoCq	NoCq	38.700	23.623	26.526	28.967	23.155	26.254	28.753	23.501	26.560	31.496
I9	1	24.351	29.675	33.712	25.118	29.188	31.891	25.203	29.209	34.896	24.599	29.300	36.882
F4		23.203	26.009	27.026	24.760	26.852	27.565	24.888	27.392	28.543	24.218	27.047	29.023
C4		NoCq	NoCq	NoCq	23.950	27.034	29.875	23.637	26.328	33.146	23.567	27.010	35.838

**Table 4:** Repeatability and reproducibility of the multiplex TaqMan real-time PCR assay.

Target Gene	Plasmid Concentration (Copies/μL)	Intra-Assay			Inter-Assay		
		Mean Ct	SD	CV (%)	Mean Ct	SD	CV (%)
*inv*	1 × 10^6^	19.205	0.024	0.126	18.948	0.433	2.285
	1 × 10^5^	22.737	0.132	0.579	22.667	0.188	0.830
	1 × 10^4^	26.555	0.123	0.462	25.803	0.674	2.610
*caf1*	1 × 10^6^	18.098	0.076	0.421	17.903	0.424	2.370
	1 × 10^5^	21.603	0.155	0.717	21.641	0.140	0.646
	1 × 10^4^	24.817	0.093	0.376	24.531	0.259	1.057
*foxA*	1 × 10^6^	17.568	0.096	0.549	17.498	0.335	1.912
	1 × 10^5^	21.262	0.167	0.786	21.170	0.131	0.621
	1 × 10^4^	24.194	0.121	0.498	24.006	0.240	1.002

## Data Availability

The original contributions presented in this study are included in the article. Further inquiries can be directed to the corresponding authors.
